# A Clinical Lecture*Abstract of lecture delivered in amphitheatre of the Virginia Hospital to the students of the University College of Medicine.

**Published:** 1899-03

**Authors:** Stuart McGuire

**Affiliations:** Richmond, Va.; Professor of Principles of Surgery in the University College of Medicine; Surgeon to St. Luke’s Home, and to the Virginia Hospital


### A CLINICAL LECTURE *

### 1. RESULT OF GRAFTING WHITE SKIN ON A BLACK PATIENT. 2. BASSINIS'S OPERATION FOR THE RADICAL CURE OF INGUINAL HERNIA. 3. EXTERNAL PERINEAL URETHROTOMY FOR STRICTURE OF THE URETHRA.

BY STUART McGUIRE, M.D., RICHMOND, VA.

Professor of Principles of Surgery in the University College of Medicine; Surgeon to St. Luke's Home, and to the Virginia Hospital.

### WHITE SKIN GRAFTED ON BLACK PATIENT.

Before beginning the regular work of to-dav, I wish to exhibit a patient who illustrates the fact that *skin grafts do not always acquire the color of the individual on whom they grow*, and demonstrates the importance of matching the borrowed skin to the adjacent integument in cases of cosmetic work where the result of the operation is in an exposed position. Some of you will remember this negro, whose leg was amputated in the clinic over a year ago. Owing to an effort to save too much of the limb, sloughing occurred in the flaps, and a raw granulating surface resulted, over six inches in diameter. You will recollect that as soon as active suppuration ceased he was brought before you again, and the defect covered by Thiersch's method of skin-grafting. Usually skin-grafts are cut from the individual's thigh, but in this instance they were taken from the leg of a white man which had been amputated a few moments before. I remember telling you that it seemed a shame to mutilate black skin when so much white skin was going to waste, and expressing my belief, based on the investigations of Karg, that pigmentation would occur and that the white skin would gradually become black. The operation of skin-grafting was a perfect success, and the patient was discharged in two weeks with a well-healed stump. He comes back for exhibition to-day. The artificially formed skin is firm, pliable and painless, but *as white as the day it was implanted.* Fortunately, owing to its position, it is a matter of no consequence. Had it been upon the face, and had the colors been reversed, there might be a lively suit for malpractice.

### REDUCIBLE INGUINAL HERNIA.

The first case on which I will operate is one of *reducible inguinal hernia.* I have not time to discuss its anatomy, varieties or symptoms, and will confine what I have to say to its treatment.

A few years ago the patient would have been advised to wear a truss, and to resign himself to the inconvenience of the

*Abstract of lecture delivered in amphitheatre of the Virginia Hospital to the students of the University College of Medicine.

apparatus, and to subject himself to the danger of strangulation rather than submit to an operation which was attended by danger, and which gave but little prospect of a permanent cure. To-day the perfection of aseptic technique and the introduction of Bassini's method of operating have so reduced the risk to life and so increased the chances of a radical cure, that the opinion of the profession has changed, and those best qualified to advise earnestly urge operative intervention in such cases.

In a normal condition, the inguinal canal contains only the spermatic cord. In inguinal hernia, it contains both the spermatic cord and the hernia sac. An operation aims to constrict the canal so as to prevent the escape of the hernia, but not to obliterate the canal so as to prevent the passage of the cord.

The reason the old operations were failures was because surgeons at first devoted all their efforts to attempts to narrow the external abdominal ring, and when they finally realized the necessity of splitting open the canal and attacking the internal ring, they failed to appreciate or correct the anatomical defect which normally exists in the posterior wall of the canal, due to the absence of all muscular tissue.

*Bassini's operation* meets all indications, and the results are all that could be desired. It is simple, safe and effective, and is almost universally adopted. The only point upon which differences of opinion exist is the character of suture to employ. Some surgeons use silk; some Kangaroo tendon; some chromicized catgut, and some silver wire. I will describe the different steps of the operation as I excute them.

The patient is prepared as for an abdominal section, the diet being regulated, the bowels emptied, and the skin made active. The site of the operation is carefully sterilized by shaving, by the application of a soap poultice, by vigorous scrubbing, and by the use of alcohol and bi-chloride of mercury.

The patient is anesthetized, and an incision about three inches long is made parallel with and immediately over the inguinal canal. The cut is deepened until the aponeurosis of the external oblique is exposed, which is recognized by the white glistening surface and the direction of the fibres.

The external abdominal ring will be found at the lower angle of the incision, with the spermatic cord and hernial sac passing through it. The internal abnominal ring will be found at the upper angle of the wound, covered by the aponeurosis of the external oblique muscle. A grooved director is next inserted through the external ring, and carried upward and outward to the internal ring, and the anterior wall of the inguinal canal incised, thus exposing its entire length. The cord and sac are lifted out and carefully separated one from the other. The sac is opened, its contents returned to the abdominal cavity, and the neck ligated with fine silk at the internal abdominal ring—a point flush with the general peritoneum. The empty sac is then amputated a short distance below the ligature, stripped out of its bed and removed.

Now comes the insertion of the sutures, the distinctive feature of the operation, by which the internal opening is changed, a new posterior muscular wall is made, the external opening is narrowed, and the obliquity of the canal is restored. The cord is held to one side by an assistant, and a curved needle, threaded with chromicized catgut, is passed through the conjoined tendon of the internal oblique and transversalis muscles well up towards its attachment to Poupart's ligament. The needle is then carried under the cord and through Poupart's ligament. The suture is tied, and results in bringing the tissues snugly around the cord and placing it in the angle of juncture of the two structures named. The edges of the conjoined tendon and Poupart's ligament are next united by a continuous catgut suture from the new-formed internal ring to their common insertion in the public bone. The cord is now placed upon the posterior muscular wall which has been formed, and the divided edges of the aponeurosis of the external oblique are united over it by interrupted sutures of catgut. The skin is then closed by a subcuticular suture. No drainage is employed. The wound is dressed with a pad of gauze and cotton, held firmly in place by a spica bandage. It will be left undisturbed until healing is complete.

### IMPERMEABLE STRICTURE OF URETHRA.

The next case is that of a man with *impermeable organic stricture of the membranous portion of the urethra*, and the operation which I will do for the relief of the trouble is called *external perineal urethrotomy without a guide.* In order that you may understand the nature of the case and comprehend the steps of the operation, I will devote a few moments to the discussion of the condition.

Stricture of the urethra is an abnormal diminution in the lumen of the canal. It may occur at one point, and then is called single; it may occur at several points, and then is called multiple. It may be due to temporary causes, as irritation from drugs, sexual excesses or abnormal conditions of the urine, and then is called spasmodic stricture. It may be due to permanent causes, as the formation of cicatricial tissue from inflammation due to traumatism or urethritis, and then is called organic stricture.

An organic stricture may occur in either the spongy or membranous portion of the urethra; its gravity is in direct proportion to its distance from the meatus. It may be of large calibre, and permit the passage of an instrument, and is then called a permeable structure. It may be so tight and tortuous that it will not permit the passage of even a fine filiform bougie, and then is called an impermeable stricture.

Organic strictures are treated by one of two methods: by dilating or by cutting. Dilatation can only be practiced when the calibre of the stricture is sufficient to permit the passage of a fair-sized steel bougie. It is carried out by introducing a progressively larger instrument at each sitting, and is successful only after long treatment. Dilatation acts not only by mechanically stretching the stricture, but also by promoting absorption of the cicatricial deposit. The method is free from danger if conducted under antiseptic precautions; does not confine the patient to bed or prevent him from following his usual occu[illegible]ion, and hence should always be tried in suitable cases before resorting to more certain but more radical measures.

If it is decided to cut a stricture, the operation can be done in one of two ways: by internal or by external urethrotomy. In internal urethrotomy, the stricture is cut from within the urethra by passing Otis' or Maisonneuve's urethrotome through the stricture, and then bringing a concealed knife in contact with the cicatricial tissue.

In external urethrotomy, the stricture is cut from without by making an incision through the skin immediately over the stricture, and deepening the wound until the scar tissue is divided and the urethra opened. In this operation, when the stricture is permeable, an instrument is introduced from the meatus to the bladder—thus locating and fixing the urethra—and the operation is said to be done with a guide. When the stricture is impermeable, and an instrument can not be introduced, the urethra must be found by careful and often tedious dissection, and the operation is said to be done without a guide.

The advantage of the internal method is the rapidity of recovery; the disadvantage is the danger from hemorrhage, due to the division of an inaccessible vessel. The advantage of the external method is the certainty of results and the ability to control bleeding by direct hemostasis; the disadvantage is the duration of confinement to bed.

The indications for the selection of the method of operating in a given case vary with different surgeons. My rule is a simple one. Internal urethrotomy in strictures of the spongy or pendulous urethra. External urethrotomy is strictures of the membranous or deep urethra.

The patient before you has stricture of the deep urethra, the result of specific urethritis. Another surgeon has performed internal urethrotomy on him three times within the past year. His failure to give relief has been in the selection of the method, not in the execution of the operation. The patient is now only able to void his urine drop by drop, and his suffering is intense.

I decided, as soon as I examined him, to do an external perineal urethrotomy, and for the past week I have made daily attempts to pass an instrument through the stricture, to act as a guide in the operation, but without success. Some authorities state that no stricture is impermeable that permits the escape of a drop of water, and that an impassable stricture at one end of a bougie may mean a lack of skill and patience, at the other. I can not subscribe to this view, as within the past year I have had four other cases in which retention of water was not complete, and in which repeated, prolonged, and conscientious efforts to enter the bladder failed. In all four cases, I did successfully what I propose to do to-day—the operation of external perineal urethrotomy without a guide.

The patient being anæsthetized, placed in the lithotomy position, and the site of the operation thoroughly cleaned, a sound of moderate size is passed down the urethra until it reaches the face of the stricture. The handle is then turned so that the point of the instrument projects in the perineum. An incision about an inch and a half in length is then made directly down upon the point of the sound, and the urethra is opened. Silk ligatures are passed through each margin of the cut urethra, and lateral traction being made by them below, and vertical traction being made by the tip of the sound above, the wound is opened as a triangle along whose base the lost urethra must be found and restored. All bleeding is carefully arrested by torsion or ligation, and a systematic search begun for the tract, every possible channel being explored with a probe or filiform bougie. At length, apparently more by luck than skill, the proper route is found, a grooved director is passed into the bladder and the stricture cut with a bistoury. The finger is then introduced into the bladder to demonstrate the re-establishment of the channel and to insure the complete patency. A catheter is passed into the bladder for temporary drainage and the wound is lightly packed with gauze.

The drainage is removed at the end of forty-eight hours. For a week or ten days the urine will escape from the incision, but the cut soon becomes filled with granulations, and the continuity of the urethra is restored. Here, as in all other operations for stricture, the results attained must be maintained by the regular introduction of a large steel bougie.

At the next clinic the patient will be brought before you again and a sound passed.—*Virginia Medical Semi-Monthly.*

